# Concise Review: Salivary Gland Regeneration: Therapeutic Approaches from Stem Cells to Tissue Organoids

**DOI:** 10.1002/stem.2455

**Published:** 2016-07-15

**Authors:** Isabelle Lombaert, Mohammad M. Movahednia, Christabella Adine, Joao N. Ferreira

**Affiliations:** a Department of Biologic & Materials Sciences, School of Dentistry, University of Michigan, Ann Arbor, Michigan, USA; b Biointerfaces Institute, North Campus Research Complex, University of Michigan, Ann Arbor, Michigan, USA; c Department of Oral & Maxillofacial Surgery, Faculty of Dentistry, National University of Singapore, 119083, Singapore; d Department of Oral & Maxillofacial Surgery, Faculty of Dentistry, National University of Singapore, Singapore; e Department of Oral & Maxillofacial Surgery, Faculty of Dentistry, National University of Singapore, Singapore

**Keywords:** Salivary gland, Radiation therapy, Salivary hypofunction, Xerostomia, Regeneration, Transplantation, Stem cells, Organoids

## Abstract

The human salivary gland (SG) has an elegant architecture of epithelial acini, connecting ductal branching structures, vascular and neuronal networks that together function to produce and secrete saliva. This review focuses on the translation of cell- and tissue-based research toward therapies for patients suffering from SG hypofunction and related dry mouth syndrome (xerostomia), as a consequence of radiation therapy or systemic disease. We will broadly review the recent literature and discuss the clinical prospects of stem/progenitor cell and tissue-based therapies for SG repair and/or regeneration. Thus far, several strategies have been proposed for the purpose of restoring SG function: (1) transplanting autologous SG-derived epithelial stem/progenitor cells; (2) exploiting nonepithelial cells and/or their bioactive lysates; and (3) tissue engineering approaches using 3D (three-dimensional) biomaterials loaded with SG cells and/or bioactive cues to mimic in vivo SGs. We predict that further scientific improvement in each of these areas will translate to effective therapies toward the repair of damaged glands and the development of miniature SG organoids for the fundamental restoration of saliva secretion.

## INTRODUCTION

### A Place for Cell-Based Therapies

Irreversible SG hypofunction and its associated symptoms, termed xerostomia, are a hallmark of several systemic diseases, such as Sjögren′s syndrome, granulomatous diseases, graft-versus-host disease, cystic fibrosis, uncontrolled diabetes, human immunodeficiency virus infection, thyroid disease, and late-stage liver disease [1]. Hyposalivation is also the most significant long-term complication for more than 550,000 patients that are annually diagnosed with head and neck cancer (HNC) globally and for whom radiation therapy (RT) is the main treatment [2–4]. Saliva is required for digestion, lubrication, oral homeostasis and protection against a variety of microbial and environmental hazards. Thus, a lack in saliva production can cause various life-disrupting pathological events. Rampant caries, painful mucositis, oral fungal infections, taste loss, speech deficits, and difficulty in swallowing are just a few examples of events that greatly impair patients oral and systemic health [3].

Current preventative therapies, such as surgical SG relocation outside the radiation field [5] or use of free radical scavengers [6] are challenging or not always effective. Using advanced SG-sparing intensity-modulated radiation therapy (IMRT) can still result in xerostomia, even though partial improvement of salivary secretion may occur [2, 3, 7]. This functional outcome of IMRT is correlated to each HNC patient′s personalized radiation treatment plan that all or not may affect specific regions harboring epithelial stem/progenitor cells [8] and its unique environment.

**Figure 1.**
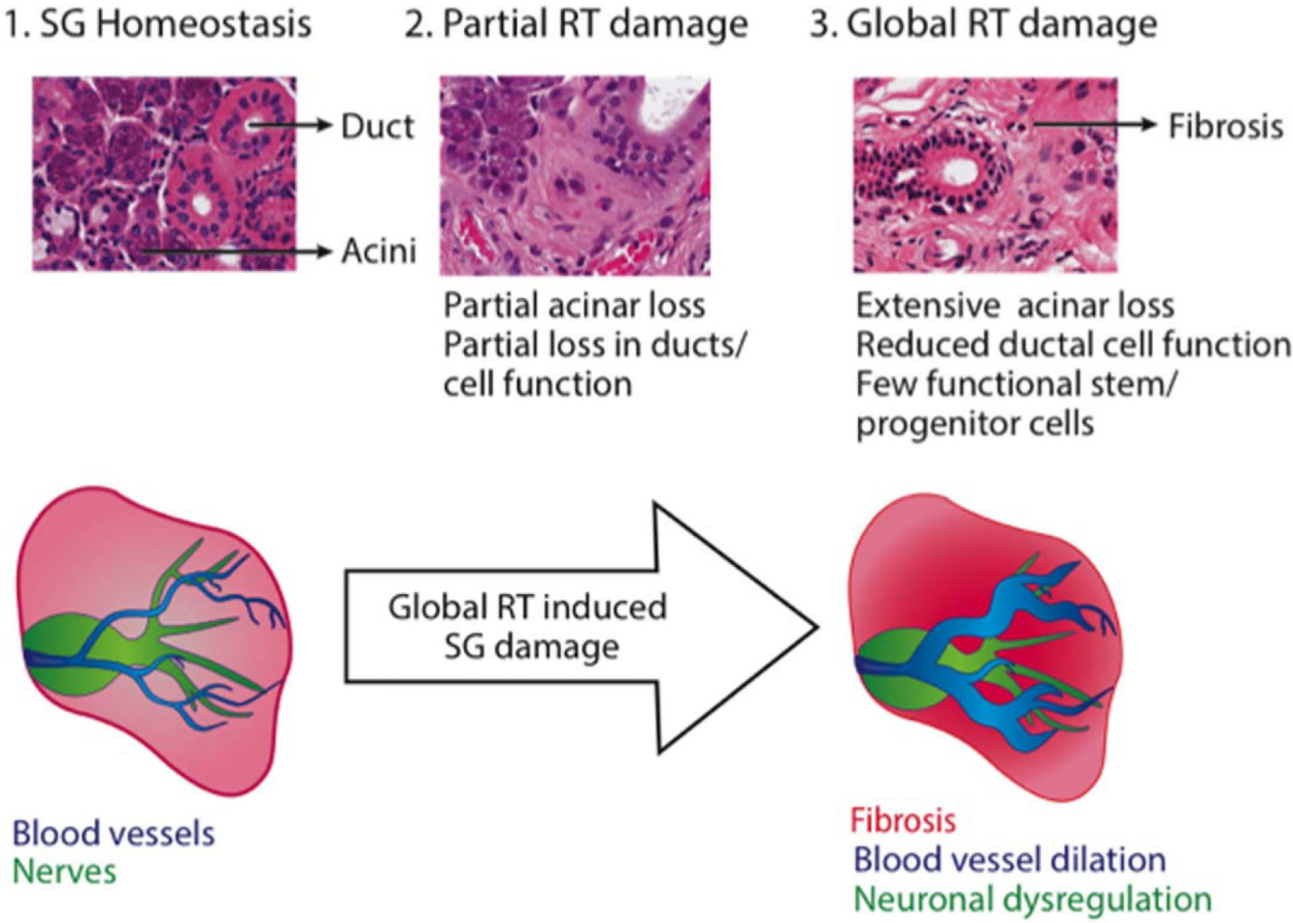
Different stages of damage in salivary glands evoked by radiation therapy (RT). (1) During salivary gland (SG) tissue homeostasis, glands are innervated and vascularized to support the epithelial compartment that consists of ductal and acinar cells. (1) Upon partial RT damage, parts of the gland are mild to moderately affected by RT (depending on the species), including the acinar compartment. (3) When RT damage globally affects the SG, massive fibrosis with varying degrees of inflammation can be observed with extensive loss in acinar and stem/progenitor cells. The irradiated glandular tissue is further marked by reduced endothelial function and neuronal dysregulation. Abbreviations: RT, radiation therapy; SG, salivary gland.

The epithelial compartment of SGs consists of nearly 80% saliva secreting acinar and 20% saliva transporting/modifying ductal cells. When SGs are in the radiation field, radiation damage occurs to these epithelial cells as well as surrounding blood vessels and nerves [4, 9]. While radiation-induced leakage of granules was long considered to be the cause of acute loss of saliva secretion, it couldn′t fully explain why proteolytic enzyme leakage was not accompanied with immediate epithelial cell loss [10]. Main causes of acute radiation damage were later credited to disturbed signal transduction pathways on the cell membrane. Irreversible damage to muscarinic receptor stimulated watery secretion [11] and dysfunction in water channels like Aquaporin 5 [12] more likely explain the high and early radiosensitivity effects. Thereafter, late to very late RT glandular dysfunction responses are due to parenchymal cell loss by apoptosis, and varying degrees of inflammation and fibrosis [10]. Even though most ductal epithelia remain morphologically, it is clear that their cellular function is impaired to some extent after RT, based on the reported decrease in protein expression of signaling receptors and structural cytokeratins [13]. Late-response effects further correlate with damage to the surrounding microenvironment by noticeable blood vessel dilation and function loss [14]. More recently, reduced parasympathetic nervous function was also suggested to be part of late post-RT effects [15, 16]. As nerves and blood vessels aid in epithelial cell repair post-RT, the combined radiation damage to acini, ducts, nerves and blood vessels, and development of fibrosis further obstructs normal gland regeneration (Fig. 1) [4].

**Figure 2.**
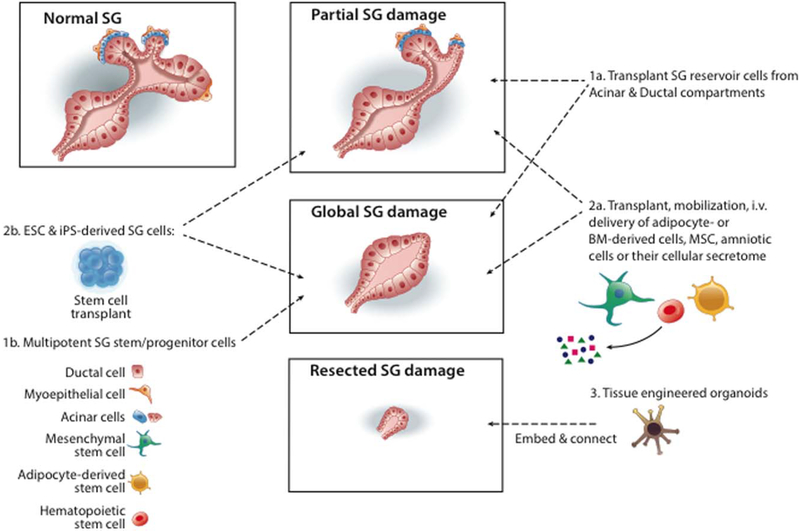
Proposed therapies to regenerate radiated salivary glands (SGs). Different epithelial cell types are maintained during homeostasis: ductal (intercalated, striated, granular convoluted tubule, and excretory), myoepithelial, and acinar cells. When glands are partially or globally injured, epithelial cells can undergo apoptosis and/or become functionally damaged. (1a) Reservoir cells of acinar and ductal compartments could then be transplanted post-radiation to locally repair the epithelia. (2a) Similarly, adipocytes, bone marrow (BM)-derived cells, mesenchymal stem cells (MSC) and/or amniotic cells can be transplanted, mobilized or intravenously (i.v.) delivered to aid in repair mechanisms. They can either participate in the formation of glandular cell types or stimulate radiation-surviving cells with their cellular secretome. (1b) After global SG damage, transplantation of multipotent SG specific epithelial stem/progenitors were shown to functionally and morphologically repair the tissue. (2b) Transplants of embryonic stem cells (ESC) and iPS (induced Pluripotent Stem cells) have also been explored to replace lost glandular cell types. (3) When SG are resected, in vitro tissue engineered organoids can be embedded in extracellular matrix and/or biomaterials and placed in the glandular bedding to connect with remaining tissue residues. Abbreviations: BM, bone marrow; MSC, mesenchymal stem cells; ESC, embryonic stem cells.

The use of artificial saliva substitutes provides temporary relief of xerostomia [4], and the administration of systemic sialogogues such as Pilocarpine increases saliva secretion, but their efficacy relies on the amount of remaining functional SG cells [18]. As such, HNC patients with extensive SG damage still await treatments to permanently restore salivary function. Due to our improved understanding of tissue morphogenesis and how (partially) damaged cells can be re-activated or replaced, several cellular and tissue-based therapies have been proposed to repair damaged SGs and/or generate new SG tissues (Fig. 2) [19, 20]. Despite cellular differences within the three major SGs (parotid, submandibular, and sublingual) are present, predominantly in the ratio of serous and mucous acini and potentially in their unique set of progenitors, researchers mainly focused their SG regenerative studies on submandibular and parotid glands. However, we propose that the following therapies may be applicable to all major glands. These can be grouped in the following categories:

Autologous epithelial stem/progenitor cell transplantation: prior to RT, cells can be isolated from SG biopsies, potentially in vitro cultured and cryopreserved during RT, and transplanted into the irradiated gland post-RT to replace functionally damaged and/or lost cells.

Application of non-epithelial specific cell types and/or their bioactive lysates: (a) to trigger paracrine regenerative effects on remaining SG cells after SG damage or (b) to generate new SG-specific cells or (c) transplant bioengineered SG tissue into the gland space using cells cultured with biomaterials and/or growth factors.

These proposed therapies have been tested in rodent models and recent outcomes will be highlighted in the following sections. Despite cellular differences are present between the three major SGs, we classified them

## A VARIETY OF CELL-BASED THERAPIES TO CHOOSE FROM

### Autologous Transplant of SG Epithelial Cells

As mentioned earlier, partial gland loss-of-function can in certain situations be spontaneously recovered post-IMRT [21]. This lead to the hypothesis that endogenous SG cells can participate in organ repair, and thus that cell transplants could potentially be useful to regenerate severe loss-of-function.

The first proof-of-concept study for transplanting autologous SG cells to increase salivary function was carried out in rodents and used epithelial cells expressing the cell surface receptor KIT (c-Kit, CD117). Only as few as 100–300 KIT+ cells were required to generate new acinar and ductal structures and to significantly improve organ function after radiation[19]. This research demonstrated that mouse SGs contain cells with stem/progenitor properties that when transplanted could maintain themselves and differentiate into multiple specialized SG cell types.

Further studies using transplantation of murine KIT+ sub-populations (KIT+ CD24+, KIT+ CD49f+, KIT+ CD24+ CD49f+, KIT+ CD24+ SCA1+), illustrated that KIT+ cells possess different levels of stem/progenitor activity, with KIT1 CD24+ (CD49f+/SCA1+) cells reported to be the most potent [13, 22]. These cells are likely located within the major ducts of the central SG region where the highest stem/progenitor cell number resides [8]. Thus, KIT+ cells have potential for future cell therapy applications, particularly because they are present in human SGs [23] and can be isolated and cultured ex vivo [24]. A very recent ground-breaking study[25] has further supported the clinical use of enriched KIT+ subpopulations. Researchers were capable of rescuing hyposalivation in an in vivo mouse model with at least 500 human KIT+ SG cells per gland [25]. Moreover, regulators of the Wnt pathway were found upregulated in the SG tissues post-transplantation. The same research group showed earlier that the activation of the Wnt pathway is essential to drive the self-renewal of murine SG stem/progenitor cells in vitro [26].

Yet, the use of techniques such as genetic lineage tracing in mice, the application of DNA labels to mark label-retaining quiescent cells, in vitro floating sphere assays (or salispheres), and two-dimensional (2D) or three-dimensional (3D) cultures of both human and rodent SG cells revealed the existence of multiple stem/progenitor-like cells in the SG. These stem/progenitor cells can be identified and isolated based on the expression of a set of proteins and/or enzymes, such as cell surface receptors and cytokeratins (Table 1). Interestingly, these stem/progenitor cells appear at different times during organ development and may compensate for each others cell loss to allow proper organ formation [49]. Even during adult SG homeostasis, multiple reservoir cell types in compartments, such as ducts and acini, harbor high mitotic capacity and the ability to self-duplicate, that is, maintain and/or expand themselves [36, 50, 51]. However, from studies on SGs and other branching organs (reviewed in [52, 53]), it becomes clear that these compartmental reservoirs of stem/progenitor cells that regulate homeostatic maintenance may respond differently to tissue damage and/or become plastic by contributing to a cell population they normally do not form.

Thus, even though KIT+ cells as well as CD24+/CD29+ epithelial cells have been shown to restore hyposalivation in vivo ([13, 19, 22, 54], we can not rule out that other cell types are not able and/or are more potent to regenerate SGs. Table 1 summarizes different cell markers that were classified with stem/progenitor potential, but majority were not fully tested yet for their regenerative capacity in RT clinical settings.

Additionally, depending on the location and level of RT-induced damage in the SG, different stem/progenitor cells could potentially be used for repair. A recent study [8] revealed that a specific region within the gland is more sensitive to radiation than others, and that radiation to this area reflects in severe saliva loss and tissue damage. When the 50% of cranial region of the SG was radiated, the entire gland degenerated including the shielded caudal 50% [8]. In contrast, damage remained restricted to the 50% caudal region when only this part was being radiated. This suggests that once multipotent stem/progenitor cells, which are proposed to be located in a cranial sub-volume, are lost other cell types are not able to compensate and repair the gland [8]. However, when cranial stem/progenitor cells remained unaffected they were able to maintain this area of the gland functional. As such, it now becomes speculative whether different cell types could be used in each scenario. For example, while transplantation of multipotent stem/progenitor cells becomes preconditioned when the entire SG is damaged, less potent cells and/or multiple compartmental reservoir cells could be applied for local caudal SG repair [8]. Even acinar cells, which were long assumed to be permanently differentiated and post-mitotic, could now be considered for SG cell therapy as they can self-duplicate after damage in post-duct ligation [36], partial SG excision [55], post-chronic sialadenitis [56] and possibly post-RT conditions to locally repair and maintain the secretory compartment. Most interestingly, SG repair is not only driven by transplanted cells, but also by the remaining endogenous stem/progenitor cells [25]. Radiation can induce stem/progenitor cell dormancy in vivo [41, 57], and thus these cells can be locally activated with the appropriate stimuli. As such, any type of transplanted epithelial cell could enhance local endogenous repair if the appropriate stimuli are produced and a dormant stem/progenitor cell is present nearby.

However, from a clinical standpoint there may be limitations to autologous cell therapy since SGs from aging patients contain fewer stem/progenitor cells [24, 58]. This implies that more stem/progenitor cells (than those obtained in the pre-RT biopsy) may be required for organ repair. Recent efforts to increase the number of KIT+ cells ex vivo using growth factors [59] or Aldehyde dehydrogenase-3 (ALDH3) activator [28] may be useful, although, the absolute cell number required for functional regeneration of the human gland remains unclear. Alternatively, non-SG cells may be considered to address this limitation, as outlined below.

Another caveat in developing SG cell therapies could potentially be the limited lifespan of biopsy-derived cells cultured ex vivo. In such cases, methods to cryopreserve and store these progenitors from biopsies have been developed. Neumann and others [60] established a stem cell banking model where SG CD49f+ CD29+ cells were cryopreserved for up to 3 years without affecting their genetic or functional stability, validating that cryopreservation could be part of a cell therapy option in the near future.

In conclusion, multiple research groups have shown that rodent SG-specific epithelial cell transplantation is a feasible approach to repair irradiated SGs. Future research studies will determine whether human SG cells behave in a similar manner in ex vivo and in vivo assays [25]. Although success has been achieved with epithelial KIT+ cells in rodents, currently, other more multipotent stem/progenitor cell candidates and/or compartmental reservoir cells can be explored. Alternatively, in clinical scenarios where autologous SG cell numbers are low, we may need to take advantage of the regenerative capacity of non-SG cells, as discussed in the next section.

### Nonepithelial Cell Types and Bioactive Lysates

There are many reports on the beneficial effects of non-SG and/or non-epithelial cells to regenerate irradiated SGs. These studies include Bone Marrow (BM)-derived cells [14, 61–63], BM-derived mesenchymal stem cells (MSC) [64], human adipose-derived MSCs [65–68], SG-derived MSC-like cells [32,69], amniotic cells [70, 71], embryonic stem cells (ESC) [72], and induced-pluripotent stem cells (iPS) [73].

Despite a proposed differentiation of BM-derived cells and MSCs into SG acinar cells is observed in vitro, their actual contribution to epithelial differentiation in vivo is not clear and disputable. Their beneficial action may primarily occur via paracrine pro-survival/proliferative effects on remaining epithelial stem/progenitor cells and surrounding environmental cells. For example, transplantation of G-CSF/FLT3/SCF-mobilized BM-derived cells [14] not only improved saliva production by inducing epithelial repair but also increased microvessel density, which consequently led to better blood perfusion. Similarly, adipose-derived MSCs diminished acinar cell apoptosis as well as reduced fibrosis [67], and both BMMSC as SG-derived mesenchymal-like cells exerted immunosuppressive activities [69].

The beneficial potential of these paracrine effects led investigators to explore the addition of the bioactive components, also called “soup,” secreted by these adipose and BM-derived cells to repair SGs who underwent RT [43, 74]. The exact content of the bioactive components remains elusive to date, but several potential contributing signaling pathways have been identified. Studies using systemic growth factor delivery or genetic overstimulation of specific signaling pathways suggest that KGF (or FGF7) can increase stem/progenitor cell numbers in vivo post-RT [41, 75]. A similar role was attributed to WNT/β-catenin [47, 48] and Sonic Hedgehog (SHH) signaling [42] in post-RT and post-ductal ligation settings. Also treatment with EGF, IGF1, FGF2 [39, 40, 45], IL6[44], ALDH3 [37], or EDA activators [38] reduced cell apoptosis and promoted proliferation (Table 1). Even post-radiation treatment with hormone Melatonin can decrease oxidative stress and lipid peroxidation in SGs [46]. Another putative activator for inducing acinar differentiation may be the NOTCH signaling pathway [76, 77], even though its beneficial action in vivo post-RT has not been confirmed yet. All these signaling factors are summarized in Table 1.

Since multiple factors (e.g., GM-CSF, VEGF, IL6, and IGF1) are found in “soups,” the anti-apoptotic and pro-proliferative cues can thus aid not only in epithelial but also in microenvironmental repair [43]. Moreover, intravenous “soup” administration may be all that is required to clinically improve saliva production as this delivery route appears to be as effective in rodents [43]. However, it remains to be evaluated whether the “soup” strategy will work as efficiently in every patient. Similar to the clinical efficacy of Pilocarpine administration in RT-induced xerostomia settings [78], the “soup” strategy relies on the amount of remaining SG cells. Thus, clinical successes will depend on the remaining cells that need paracrine stimulation and whether these stimuli are present in the “soup.” While angiogenic factors have been described to be present in certain “soups,” it is not clear yet whether neurotrophic factors are. Neuronal cells, such as the ones from the parasympathetic nervous system, aid in epithelial regeneration post-RT [15, 22] and thus, if required, neurotrophic factors such as Neurturin or Glial cell-Derived Neurotrophic Factor could potentially be (co-)delivered to radiated SGs via retrograde ductal or intraglandular injections.

While BM-derived cells and MSCs might not efficiently differentiate into SG cells, other pluripotent cell types such as ESCs and iPS cells can be explored to provide new pools of SG-specific cells. As such, SG secretory cells were already generated from ESCs [72]. This study used 3D co-culture of mouse ESCs with a human SG-derived fibroblast environment to initiate expression of SG-related markers. While the ESC-derived SG-like cells survived post-RT SG transplantation, it is still unclear whether they functionally regenerate the tissue[72]. If these cells possess genomic stability and lack oncogenic potential, both ESC [72] and iPS-derived SG cells [73] can serve as an additional cell-based therapy.

### Tissue Engineering Strategies to Generate Sg Organoids

SG tissue engineering requires three essential components:(1) cell-cell contacts; (2) cell contacts with extracellular matrix (ECM) proteins, and (3) a biocompatible and biodegradable 3D scaffold that can hold these components together [79].

Many scaffolds have been proposed, which are porous and either biologic (e.g., collagen, fibrin, silk, chitosan, alginate, hyaluronic acid (HA)) in origin or synthetic biocompatible biomaterials (e.g., poly-glycolic acid, poly-lactic acid, poly lactic-co-glycolic acid (PLGA), and polyethylene glycol), and/or mixture of both. Depending on its biodegradability, porosity, stiffness and strength, scaffolds promote cell adhesion, migration, and/or differentiation [80]. Ideally, engineered scaffolds should structurally and functionally resemble the native SG ECM architecture (reviewed in [81]).

While there are many new scaffolds being generated, researchers must implement aspects of SG organogenesis, branching morphogenesis and homeostasis to initially form 3D miniature tissues, termed organoids. A summary of currently used human cell-based models with translational potential is presented in Table 2.

A long-standing hurdle in the field has been the long-term growth and maintenance of specific acinar cell protein expression, as well as their cell polarity and secretory function. Monolayer cultures, that is, 2D culture, of primary acinar cells cause loss of biological functions including, acinar-specific protein expression (α-amylase, cystatin C, transmembrane protein 16A—TMEM16A, sodium-potassium-chloride cotransporter—NKCC1, and aquaporin 5—AQP5), granule formation, calcium mobilization, transepithelial resistance, and polarized amylase secretion after β-adrenergic receptor stimulation. Gaining control of these biological functions appears to be related to specific media components and ECM products. High calcium concentrations (0.05 mM) provide optimal acinar growth and maintenance of polarization [85], and without addition of ECM proteins the maintenance of acinar cells and formation of organoids will be limited. For example, pure amino acid non-ECM containing PuraMatrix peptide hydrogels hardly maintained SG cells [86], but mucin-secreting cells were easily grown for up to 1 month on natural fibronectin-coated silk fibroin scaffolds. Interestingly, 3D scaffolding itself induced seeded cells to produce significantly more native ECM components than in 2D cultures, which further supports more appropriate cell differentiation and polarization. The observation that parotid cell cultures were better maintained on these silk fibers compared to submandibular cells also indicated that each gland cell-type might require a unique ECM-coated scaffold.

It is also important to note that each ECM differently impacts cell polarization, differentiation, lumenization, and tight junction formation. PLGA nanofibers coupled with laminin-111 and chitosan functional units demonstrated that laminin-111 tends to promote mature SG epithelial tight junctions and apico-basal polarization, but conversely, chitosan antagonizes this process [87]. Encapsulating human SG cells in human-compatible HA hydrogels with recombinant Perlecan IV domain not only induced cell organization into proliferating spheroid structures, but also formed larger acini-like structures with a central lumen that were maintained long-term in vitro [88]. In cases where there is a reduction in the assembly of tight junctions (ZO-1 expression) [89], which are needed for uni-directional flow of saliva, one can overcome this by generating lithographically-based micropatterning curved “craters.” These craters mimic the physical structure of the basement membrane, and thus increased surface area allowed for better apico-basal polarization and differentiation of SG epithelial cells [90].

Apart from generating and maintaining proper cell types, engineered SGs further require formation of branching structures. Chitosan appears to facilitate SG branching by regulating production of basement membrane components[91], and small branching organoids could also be formed in Collagen type I and/or Matrigel [19, 24, 25, 31, 82, 83, 92]. While many positive results were obtained with Matrigel, its components are not xeno-free as it contains basement membrane proteins secreted by mouse sarcoma cells, and therefore, its use is not consistent with current Good Manufacturing Practice regulations by the US Food and Drug Administration (FDA). One alternative is to use the native organ-specific ECM that can be obtained by decellularizing tissues with detergents and then reseeding primary cells onto the gland ECM structure, as accomplished for the rat submandibular SG [93].

Recent advances have also been directed to develop more functional organoids. These efforts include combinations of linked ECM peptides and the development of controlled drug or growth factor releases from scaffolds. These can then be seeded with cells to direct differentiation and branching, with or without various SG cell types. A current challenge remains to let bioengineered tissues grow in size and properly connect with remaining cells in the transplanted area. Efforts toward this goal have recently been initiated in a mouse and rat model [84, 94]. HA-gels with primary human cells were maintained and responded to neurotransmitters when integrated in the area of resected parotid glands in immune compromised rats [95]. An alternative approach showed that fetal SG cells, both epithelium and mesenchyme, within a 3D Collagen environment could be transplanted into the space of completely resected SGs. Interestingly, a suture thread was used to provide guidance for the primary duct to reconnect with the oral cavity. Future efforts will certainly be directed to using a similar approach with adult cells. Whether a similar reconnection with the remaining duct can be obtained in humans remains to be determined.

## FUTURE PROSPECTS

Remarkable progression has been made in the last decade, but a definitive therapy for SG hypofunction has not been developed due to intrinsic challenges that come with each approach. An underlying challenge is comparison of the animal models with human SGs. The biological differences between human and rodent SGs and understanding how they respond to RT requires further study but initial important steps have been taken [25]. Moreover, potential differences in development and/or regenerative strategies between the different glands (e.g., parotid, submandibular, sublingual) need to be considered for future clinical translations. Also complicating matters is the variation of RT damage that occurs in individual patients with respect to both the location and dose of RT as well as the patient′s age. However, with each discovery in the future, a range of precision medicine therapies may become available individualized to each patient. An appreciation of the strengths and limitations of each strategy as well as whether the patients have existing RT damage will determine what therapy will be designed and delivered.

Theoretically, there should be no shortage of cell types, as both SG-specific as non-SG specific cells could be used to repair the epithelial compartment and surrounding microenvironment. The paracrine effects of each cell type will aid in the repair process post-RT, and with the development of bio-active scaffolds, we should be able to generate branching SG organoids in the near future.

## Figures and Tables

**Table 1.** Summary list of suggested stem/progenitor cell markers and environmental signaling cues (cytokines, growth factors, enzymes, and hormones) currently studied for salivary gland regenerative therapies

Stem/progenitor cell markers	Cytokines, growth factors, enzymes, hormones
ABCG2 [27]	ALDH3 activator [37]
ALDH3 [28]	EDA [38]
ASCL3 [29, 30]	EGF [39]
CD24 (HSA) [22, 31]	FLX3 [14]
CD29 (ITGβ1) [31]	FGF2 [40]
CD34 [32]	FGF7 [41]
CD44 [33]	G-CSF [14]
CD49f (ITGα6) [13, 34]	SHH [42]
CD90 (Thy-1) [34]	IL6 [43, 44]
CD105 [32]	IGF1 [45]
CD117 (KIT) [13, 19, 22, 25]	Melatonin [46]
KRT5 [15, 16]	SCF [14]
KRT14 [23]	VEGF [43]
MUSASHI-1 [19]	WNT [26, 47, 48]
SCA-1 [19, 22]p75 [34]	
SOX2 [35]	
MIST1 [36]	

**Table 2.** Human cell-based therapy models already tested for the development of salivary gland 3D tissue organoids

Model features	In vivo/in vitro remarks	Limitations	Reference
hSG primary cells in 3D matrix containing Collagen and Matrigel	In vitro formation of functional and differentiated salivary components containing amylase producing acinar-like cells and ductal structures	No in vivo studies	[82]
		Xenogeneic biomaterials not suitable for clinical translation	
		No evaluation of salivary flow	
hSG progenitor cells in 3D Matrigel-based matrix	In vitro differentiation ability of hSG progenitors into epithelial-like acinar and ductal cell types	No in vivo studies	[24]
		Xenogeneic biomaterials not suitable for clinical translation	
	In vitro long-term self-renewal ability.	No evaluation of salivary flow	
hSG primary cells in serum-free conditions in Matrigel-coated dishes	In vitro 3D organization and differentiation of hSG cells into salivary cells with amylase-producing acinar components	• No in vivo studies	[83]
		Xenogeneic biomaterials not suitable for clinical translation	
		No evaluation of salivary flow	
hSG primary cells in 3D HA hydrogel	HA hydrogel supported in vivo lumen formation	No evaluation of salivary flow	[84]
	Supported viability and salivary phenotypic features of hSG progenitors in in vitro long-term cultures		
hSG primary cells in a 3D matrix containing Collagen and Matrigel	Matrigel supported in vitro expansion in long-term cultures	No in vivo studies were performed with cell-loaded matrix	[25]
	3D xenogeneic matrix supported differentiation of primary cells		
		Xenogeneic biomaterials not suitable for clinical translation	
	Injected hSG primary cells (>500/gland) induced functional rescue		

Abbreviation: hSG, human salivary gland.

## SIGNIFICANCE STATEMENT

This review covers recent advances in translating cell-based research toward pre-clinical therapies. We focus on salivary gland (SG) loss-of-function and subsequent dry mouth syndrome as caused by radiation therapy or systemic disease, although the described concepts can be translated to other injured somatic tissues. Proposed therapies include implantation of autologous tissue-specific stem/progenitor cells, non-tissue specific cells and/or their bioactive lysates (secretome); and organoid-like constructs created by cells in the presence or not of bioactive cues and three-dimensional biomaterials. These emerging approaches to repair damaged SGs are discussed herein, and evaluated on their success to restore native tissue architecture, epithelial cell polarization, ductal branching, lumen formation, directionality of secretory flow, and clinically relevant tissue functionality.

## LIST OF ABBREVIATIONS

SG: salivary gland
RT: radiation therapy
HNC: head and neck cancers
3D: three-dimensional
2D: two-dimensional
KRT: cytokeratin
FGFR2b: fibroblast growth factor receptor 2b
ALDH3: aldehyde dehydrogenase-3
BM: bone marrow
MSC: mesenchymal stem cell
BMSC: bone marrow stem cell
BM-MSC: bone marrow-derived stem cell
BM-cMSC: bone marrow clonal mesenchymal stem cell
SMG: submandibular gland
FGF: fibroblast growth factor
GM-CST: Granulocyte-macrophage colony-stimulating factor
Flt3: Fms-Related Tyrosine Kinase 3
SCF: stem cell factor
KGF: keratinocyte growth factor
VEGF: vascular endothelial growth factor
IL6: interleukin 6
IGF1: insulin-like growth factor 1
ESC: embryonic stem cells
iPS: induced pluripotent stem cells
PLGA: poly-(lactic-co-glycolic acid)
HA: hyaluronic acid
ECM: extracellular matrix
